# Analysis of spatial perception and the influencing factors of attractions in Southwest China’s ethnic minority areas: The case of Dali Bai Autonomous Prefecture

**DOI:** 10.1371/journal.pone.0285141

**Published:** 2023-06-13

**Authors:** Xiaoyan Yin, Xin Han, Taeyeol Jung

**Affiliations:** Department of Landscape Architecture, Kyungpook National University, Daegu, South Korea; Zhejiang Gongshang University, CHINA

## Abstract

As standards of material living continue to improve and urbanization advances, an increasing number of remote ethnic minority areas are becoming tourist destinations. Understanding tourists’ perceptions on a large scale is thus crucial for the development of the regional tourism industry. However, traditional research methods suffer from high costs, small sample sizes, and low efficiency, making it difficult to measure the spatial perception of remote areas on a large scale. This study constructs a research framework for spatial perception measurement of remote ethnic minority areas by collecting reviews data from Ctrip using spatiotemporal data calculation and the Geodetector model. We considered Dali Prefecture as an empirical case and analyzed tourists’ perceptions of the area’s attractions, the spatial distribution of the attractions, and the process of change in the explanatory power of their influencing factors over an eight-year period (2014–2021). The results indicated that the most visited attractions were concentrated in Dali City. The perception of humanistic resources (attractions) with historical value was the highest, followed by natural resources. The high perception of attractions was influenced by the level of tourism development, traffic accessibility and attractiveness, and had an increasing influence on tourists’ perceptions over time. Additionally, changes in the mode of transportation from road to high-speed rail played an important role in the selection of tourist attractions. Conversely, the tourists paid relatively less attention to humanistic resources (e.g., national cultural heritage protection units and traditional villages). Our study provides a basis for the measurement of spatial perception in remote minority areas and can be used as a reference for tourism development planning in Dali Prefecture, thus promoting the sustainable development of tourism in the area.

## 1. Introduction

With the rapid development of China’s economy, the country has gradually developed a tertiary industry, i.e. the tourism industry; its role in the national economy has become increasingly important [1]. In this regard, tourist attractions play a major role in creating tourism revenue [2,3]. From the perspective of tourist demands, tourist attractions present unique attributes (e.g., authenticity, sensibility or scarcity) [4,5]. As Boyd indicated, the uniqueness of a tourist attraction is often the only reason why tourists want to visit a given location [6].Over the past few decades, scholars have examined tourism attractions, mainly focusing on marketing and planning [7,8], demand forecasting [9,10], tourists’ perceptions and behavior [11,12], spatial distribution and structure [13] and factors influencing the spatial distribution of attractions [1,14,15]. In China, there have been many studies on the spatial distribution of attractions and their influencing factors, with the main objects being national A-level tourist attractions [14–16]. However, research on the spatial perceptions of attractions actually visited by tourists and their influencing factors has been limited.

The collection of data is the biggest challenge for spatial perception research. The traditional method to measure spatial perception is to recruit experts and the public for subjective evaluations of spatial perception through questionnaires, scales, or interviews [12,17,18]. However, the participants are susceptible to external interferences that may trigger biased results; there are other disadvantages such as high costs, long durations, small data size, and low efficiency, which limit the depth of related studies to a certain extent; therefore, these methods support the study of spatial perceptions of small-scale sites and do not support long-term spatial perception studies on large regional scales [19].

Recently, the development of big data from sources such as photographs containing geographical information uploaded by tourists has provided research data to analyze perception studies over long periods of time in large regional areas. However, the majority of this research has been limited to urban spaces in developed regions due to the difficulty of accessing large amounts of geotagged photographs from photo-sharing sites in remote ethnic minority areas. Therefore, this study collected Ctrip review data and used spatiotemporal data calculations and the Geodetector model to spatialize the data of attractions visited by tourists in a large area, so as to evaluate the spatial perception of tourist attractions in remote minority areas. To a certain extent, this method compensates for the lack of spatial perception research in the past, expands the scope of data collection in big data-based tourism research, and extends the research perspective of spatial perception.

In this study, Dali Bai Autonomous Prefecture (hereafter, Dali Prefecture) was chosen as the subject because it has been a popular tourist destination since the 1990s [20]. However, most tourists tend to visit well known attractions and ignore lesser known but equally significant natural and humanistic resources, leading to the uneven development of tourism in Dali. Thus, examining the causes of such a phenomenon in greater depth is necessary.

Therefore, this study aims to accomplish the following: 1) explore the types of tourism resources perceived by tourists and the differences in tourists’ degrees of perception of tourism resources over time based on the number of reviews of attractions in Dali Prefecture; 2) analyze the spatial distribution of tourists’ perceived tourism resources; and 3) evaluate the influencing factors of their spatial perception and the differences in their explanatory power over time using the Geodetector model.

The rest of the paper is arranged as follows. Section 2 presents a review of the relevant literature. Section 3 describes the workflow of the study, data sources, and methods of analysis. Section 4 presents the results of the study. Section 5 discusses the findings in relation to previous research and indicates the limitations of this work as well as directions for potential future work. Finally, Section 6 presents the conclusions of this study.

## 2. Literature review

### 2.1 Factors influencing the spatial distribution of attractions

The factors that influence the spatial distribution of tourist attractions have been extensively researched. Based on previous studies, the following four dimensions are elaborated upon—traffic accessibility, natural environment, socio-economy factors, and core resources.

#### 2.1.1 Traffic accessibility

As an important spatial element of the tourism system [21], transport reflects the convenience of tourists’ movements to or from tourist attractions [15]. Sarma noted that transport within destinations is an essential factor for determining their attractiveness as tourist destinations [22]. A related study indicated that the distance from urban areas can affect the spatial distribution of tourist attractions [23]. Meanwhile, Su found that tourism in Yunnan Province is more dependent on road transport compared to air transport and railroad transport [24].

#### 2.1.2 Natural environment

A good natural environment is the basic condition for the development of tourist attractions. Prior studies have categorized natural environment factors as altitude, waters and average temperature [1,16]. Altitude, to a certain extent, reflects the type and distribution of natural resource tourism attractions [1], which are distributed in the mountains, while humanistic resources, such as museums and ancient villages, are distributed in the plains [16]. Additionally, the rivers themselves are a natural tourism resource [1]. Moreover, suitable temperatures can promote the cultural development of a region and facilitate the formation of natural landscapes [1]. Due to Dali Prefecture’s small geographical scale and minimal temperature differences, these elements are not considered in this study.

#### 2.1.3 Socioeconomic factors

Nowadays, the tourism demand of residents has increased; the resident population determines the scale of the local tourism market and the related tourism employees, which can have an impact on the formation and distribution of tourist attractions [1]. In this regard, areas with higher incomes have relatively higher levels of tourism development and are able to provide quality public services, such as healthcare, law enforcement and public transport, which are important components of tourism [15,25,26]. Further, the proportion of the tertiary industry largely reflects the level of the development of the regional tourism [1,15].

#### 2.1.4 Core resources

Some studies have found that some specific attractions can attract several tourists [27–30]. For example, an empirical study [31] conducted in Nanjing City, China, indicated that tourists on short trips are more likely to visit primary attractions, whereas those on longer trips combine primary and secondary attractions. Simultaneously, cultural resources such as museums, historical sites and monuments are an important attraction for tourists; tourists expect to experience different cultures and understand cultural connotations during their trips [26,32,33].

### 2.2 Related studies on spatial perception

Spatial perception is the feeling and perception formed by the interaction and influence of human and environmental elements; a perception measure is the most basic and direct method of studying human–space interaction [34]. Currently, research on spatial perception is primarily based on the perspectives of urban and rural planning, geography, and sociology [35]. Earlier, due to the limitations of science and technology, research on human perception generally used interviews and questionnaires and relied on personal experience [36]. For example, McGinn’s team conducted a hands-on exploration using a telephone survey to describe the link between human perceptions, objective measurements of the built environment, and leisure, walking, and transportation activities, assessing the perception of the built environment through the lens of human emotion [37]. Jensen conducted a questionnaire survey of 632 visitors at 4 attractions in Norway and found that visitor perceptions varied significantly depending on the attraction and type of visit [12]. Li used cognitive maps and supplemented them with questionnaires to obtain participants’ perceptions of space and found that students had a high perception preference for historical spaces [38]. However, these types of studies are expensive and time-consuming and are limited to research with specific sample conditions.

In the Web 2.0 era, the world is connected through online social networking services, allowing people to share their experiences and stories with one another [39]. In the context of tourism, tourists post travel-related information, such as photos, texts, travel tips and locations, through these platforms (e.g. Ctrip and Tripadvisor) to influence other tourists’ decisions regarding different aspects of the trip, such as the selection of tourist destinations and attractions to visit [40–42]. Therefore, using this new data to quantitatively analyze spatial perception has become a new and relevant research perspective and trend.

Existing studies have primarily used online text and photo data. Text data are mainly used for satisfaction studies, landscape perception, and travel recommendations [43,44]. Zhang took ancient Chinese towns as the subject of his study, based on the resource-based view and crisp set qualitative comparative analysis method (csQCA) and found that the destination’s core resources need to complement the resources of other destinations to generate a high perceived value for tourists [44]. Koufodontis took a sample of 105 World Heritage sites and collected the corresponding 2.5 million reviews from TripAdvisor to analyze the degree of awareness or interest in the UNESCO designation that influences tourists and locals [43]. In contrast to textual data, tourists upload photos containing geographic information, and most photo-sharing websites enable “geo-tagging” in their interface, whereby the latitude and longitude of the photos can be automatically extracted [45], making it possible to analyze the spatiotemporal patterns of tourists over a wider scale [46–48]. For example, Liu used a Python script to download photos from 26 cities around the world from Panoramio and Flickr, used content analysis to classify the photos into 102 scenes and 7 perceptions, and statistically analyzed the features and spatial distribution of the perceptions [49]. Zhang, based on the geographical information of photos, visualized maps with different perceptual themes of tourists using deep learning techniques [48]. Kim used geographic photographs from the last 10 years on Flickr to study tourists’ temporal and spatial activities in aheritage park of the Association of Southeast Asian Nations to analyze how tourists perceive different historical spaces and their interest in visiting them [50].

Although the application of new technologies and data has, to a certain extent, compensated for the shortcomings of traditional research methods; most studies continue to be limited to urban spaces in developed areas; there is thus a gap in the study of spatial perception in remote minority areas.

### 2.3 Studies on the explanatory methods of influencing factors

Li proposed a multiple linear regression model-based approach to short-term urban traffic flow forecasting to cope with the time-consuming problem of traditional short-term urban traffic flow forecasting methods and explored the influencing factors affecting traffic flow and the degree of influence [51]. Wang used deep learning techniques to identify six perceptions of city streets as beautiful, wealthy, boring, depressing, lively, and safe, explored the factors influencing these perceptions using ordinary least-squares linear regression models, and obtained the visual element features that play positive and negative roles in the six perceptions [36]. Marando used a multiple linear regression model to find the factors influencing the urban heat island effect, indicating that green infrastructure elements such as urban and peri-urban forests are likely to provide ecosystem services in the Mediterranean region [52]. However, a common problem with these studies is that some of the data are spatial, with geographical locations; if global regression models are used to explore the relationships between influencing factors and dependent variables, the explanatory power is often low compared to spatial regression models, and global regressions are prone to data collinearity problems. Spatial regression divides global regression into several local regressions, each with its own bandwidth; the accuracy of these is theoretically higher than that of the global regression [53]. Essence of the Geodetector is a spatial regression model, which provides a higher degree of explanatory power compared to traditional multiple linear regression models, and the problem of collinearity in the regression is effectively avoided for the influencing factors. Therefore, there are advantages to using the spatial regression approach of the Geodetector to explore spatial perception in this study.

## 3. Data and methods

**Fig 1:**
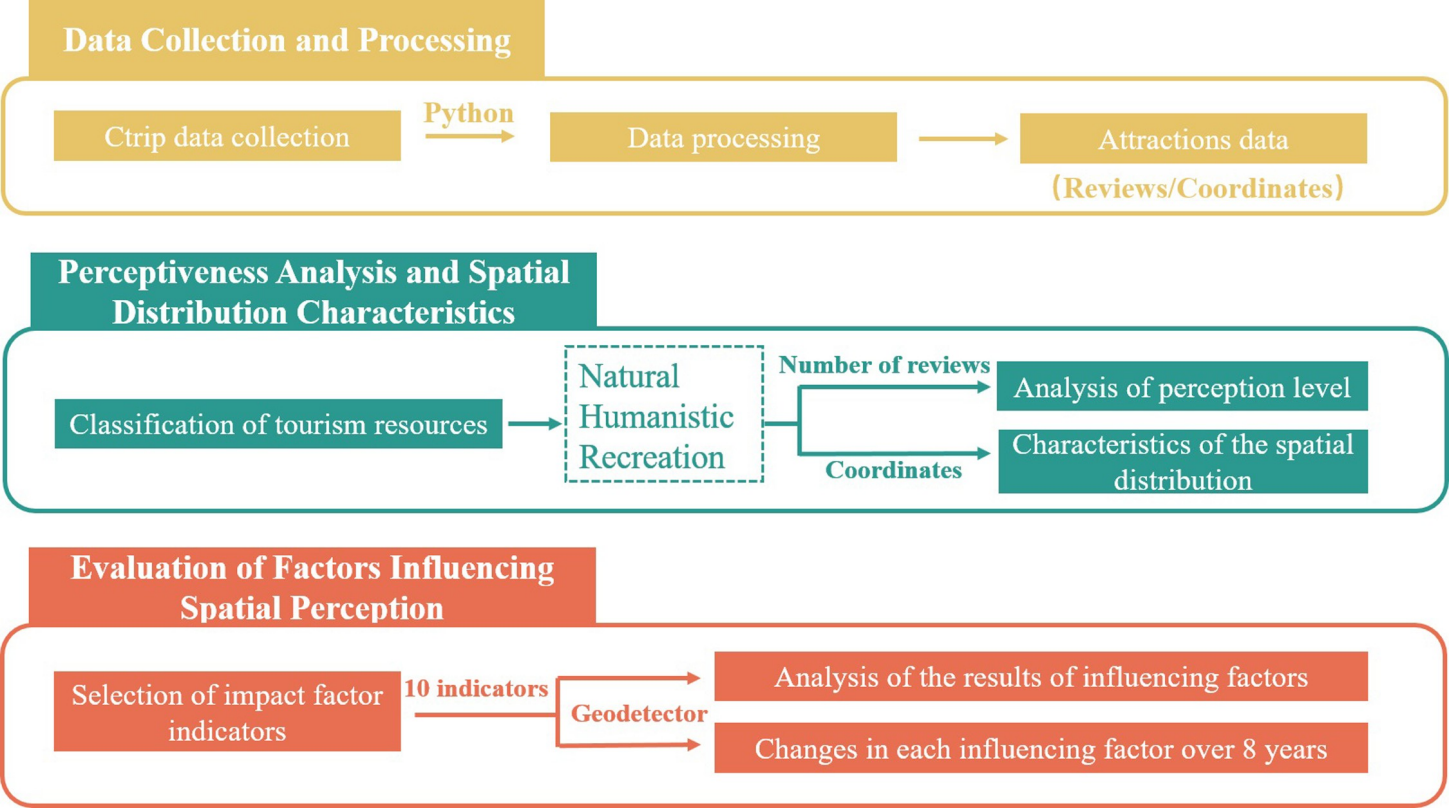
Workflow summary.

Fig 1 illustrates the framework of this study in its entirety. Firstly, Python was used to collect data shared by tourists on the travel website Ctrip (Ctrip.com) on attractions with reviews within Dali Prefecture, and the data were processed to obtain the attraction data for subsequent analysis. According to prior research, the tourism resources were categorized and the perceptions of the attractions were evaluated based on the number of reviews to obtain the tourists’ perceptions of the attractions. Then the geographical coordinates of the attractions were imported into ArcMap 10.8 to visualize the distribution and perception of the tourist attractions to clarify the tourists’ perception of the tourism resources in a large area.

Regression models were commonly used in previous studies when assessing the effects of independent variables on dependent variables, but with our data containing strong correlations between certain urban variables, the use of traditional regression models could lead to collinearity problems. Therefore, we used Geodetector to prevent the model from having weaker explanatory power due to collinearity between the independent variables and enable our model to evaluate highly correlated but slightly different city-related variables. Factor detection in Geodetector was used to evaluate whether the influencing factor indicators of spatial perception affected tourists’ perception of the attraction and analyze the differences in the explanatory power of each indicator over time.

### 3.1 Data

#### 3.1.1 Study area

**Fig 2:**
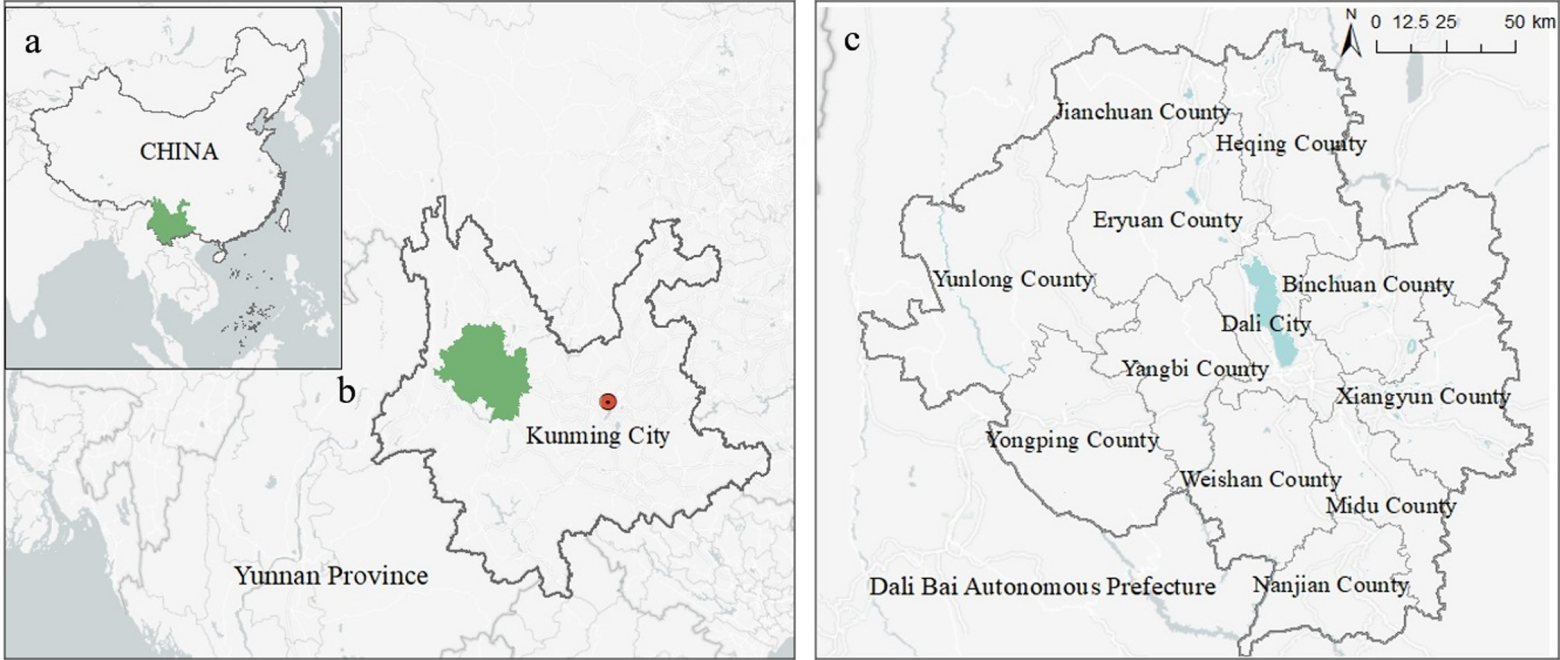
Study area (Dali Bai Autonomous Prefecture, Yunnan Province, China). Notes: a. China; b. Yunnan Province; c. Dali Bai Autonomous Prefecture. The base map images are from the China Ministry of Natural Resources(http://bzdt.ch.mnr.gov.cn/index.html). The drawing approval number is GS (2016)1593.

Dali Prefecture is located in the west-central part of Yunnan Province in Southwest China, and its capital is Dali City (Fig 2). In ancient times, Dali was the capital of the Nanzhao (738–902) and Dali (937–1254) kingdoms, which connected ancient China to South and Southeast Asia [54]. It was once a prosperous traffic hub for the Southern Silk Road and the Tea Horse Road [20].

Today, Dali refers to the Dali Bai Autonomous Prefecture, an administrative region established on 22 November 1956, consisting of the city of Dali and 11 counties, with an area of 29,459 square kilometers. It is home to 25 ethnic minorities (i.e., groups other than the dominant ethnic group in the country), with ancestors from the Bai and Yi ethnic minorities dating back to the Neolithic era [55].

#### 3.1.2 Ctrip data collection

Ctrip is the largest travel social networking site in China; it has been widely used as a research source due to its large size, frequent updates and reliable content [56]. When searching the keyword “Dali” in the Ctrip Attractions Channel, Python was used to collect the data on attractions, including basic information (name, coordinates) and text information (review time, number of reviews, contents). As of 31 December 2021, we gathered 37,485 reviews for 271 tourist attractions in Dali Prefecture.

#### 3.1.3 Influencing factor indicator selection and data sources

As in the previous literature concerning factors influencing the spatial distribution of attractions, we selected 10 indicators from 4 dimensions: traffic accessibility, natural environment, socioeconomic factors and core resources (Table 1), which were used as independent variables in this study to assess the explanatory strength of each variable for tourists visiting the attractions.

**Table 1:** Data sources and descriptions.

Factor	Variables	Explanation	Sources
**Traffic Accessibility**	Distance from a high-level highway (X_1_)	Dali Prefecture high-level highway data	*OpenStreetMap*
	Distance from a high-speed rail station (X_2_)	Dali Prefecture 2014–2021 passenger high-speed rail station data	The API of the *Gaode Open Platform* (https://lbs.amap.com/tools/picker)
	Distance from a city (X_3_)	City location data	
**Natural Environment**	Altitude (X_4_)	Altitude of each attraction	The geospatial digital elevation model
	Distance from the waters (X_5_)	Dali Prefecture waters data	*OpenStreetMap*
**Socioeconomic Factors**	Resident population (X_6_)	2014–2021 resident population data	Dali Statistical Yearbook 2014–2021
	The gross domestic product (X_7_)	2014–2021 gross domestic product data	
	Proportion of the tertiary industry (X_8_)	2014–2021 the tertiary industry data	
**Core Resources**	Distance to high-level attractions (X_9_)	2014–2021 5A and 4A attractions data	The website of the Yunnan Provincial Department of Culture and Tourism (http://dct.yn.gov.cn/)
	Distance to humanistic resources (X_10_)	2014–2021(national cultural heritage protection units	The central government portal (www.gov.cn)
		2014–2021 Chinese traditional villages	The website of Chinese traditional villages (http://www.chuantongcunluo.com)

First, regarding traffic accessibility, the high-level highway data were from *OpenStreetMap* (OSM), while the data regarding high-speed rail stations and city locations were from the *Gaode Open Platform* (https://lbs.amap.com/tools/picker). Second, the altitude data were from the geospatial digital elevation model, while the water-related data were from OSM. Third, the data regarding the resident population, the GDP and the proportion of the tertiary industry in socioeconomic factors were from the Dali Statistical Yearbook 2014–2021. Finally, the information of high-level attractions in core resources was from the website of the Yunnan Provincial Department of Culture and Tourism, and the data regarding national cultural preservation units in humanistic resources and Chinese traditional villages were from the central government portal and the website of Chinese Traditional Villages, respectively.

### 3.2 Methods

#### 3.2.1 Data processing

*Processing of data on attractions*. To ensure the accuracy of our results, we removed 1) the years with limited data in 2002–2013 [57]; 2) meaningless reviews [58]; 3) reviewers with extraordinarily active and regular reviews to avoid the influence of fake reviews [28] and 4) reviews that did not correspond to the attractions in question. Finally, 36,469 reviews of 271 attractions in Dali Prefecture from 1 January 2014 to 31 December 2021 were obtained.

*Processing of indicator data*. The resident population, the GDP and the proportion of the tertiary industry in the influencing factor indicators in Table 1 were based on data from 12 counties and cities (2014–2021), while the altitude was based on the specific altitude value of the attraction. For the remaining indicators, we used the Near Analysis Tool in *ArcMap 10*.*8* to calculate the distance from the attraction to the indicator. As the data are numerical quantities, the independent variable should be a type quantity (1–5) when Geodetector is used for analysis; therefore, in this study, the 10 variables were divided into 5 intervals by the natural breakpoint method.

#### 3.2.2 Tourism resource categories

In this study, tourism resources were categorized according to previous research. In this regard, Mizoo stated that tourism resources can be categorized into natural and humanistic resources [59], while Okamoto indicated that “natural resources” are resources that cannot be created with human power [60]. At first glance, the latter definition seems plausible, but in reality, human intervention is often required to maintain a natural order. For example, “terraced fields” appear to be a land of natural abundance, but are in fact created and maintained by humans. Thus, natural resources should be the product of a synergy between natural and human power [60].

Although humanistic resources can be recreated by human power, valuable resources, such as historical sites, should not lose their charm in the future. Conversely, various tourism resources, such as flora and fauna parks, museums and art galleries, may attract visitors now, but it is uncertain that their appeal will remain in the future [61]. While it is believed that the value of something ancient is high, the value of something recent has not been determined yet [60]. In addition, human activities, such as local customs and celebrations, can be categorized as humanistic resources [60], while facilities, such as amusement parks and sports venues, are tourism facilities set up for leisure and recreation. Hence, we categorized nature that cannot be created by human power or created by nature and human power in synergy as “natural resources,” and resources with historical value formed through human activities and those with uncertain historical value, as “humanistic resources.” Moreover, we categorized facilities set up for recreational purposes as recreation and tourism resources (Table 2).

**Table 2:** Tourism resource categories.

**Natural Resources**	Cannot be created with human power	Physiography	Hills, valleys, rock caves, landforms, typical geological zones, etc.
		Waters	Rivers, lakes, waterfalls, springs, wetlands, etc.
		Biological	Forests, grasslands, animal habitats, flora and fauna, etc.
		Natural phenomena	Skyscapes, weather, seasons
	Created by nature and human power in synergy	Terraces	
**Humanistic Resources**	With historical value	Historic sites	Human activity sites, military sites, walled city sites, etc.
		Ancient villages and towns	Ancient villages, ancient towns
		Architecture and facilities	Homes of famous people and historical architecture (non-religious architectures)
		Religious sites	Religious places of worship, temples
		Historic gardens	Gardens, historic gardens
	With uncertain historical value	Cultural venues	Museums, art galleries, cultural venues
		Modern gardens	Zoos, botanical gardens, parks
		Theme parks	Theme parks
		Featured streets	Streets, shopping streets
		Rural landscape	Villages, agricultural landscapes, rural B&Bs
	Human activities	Formed in human’s daily lives	Local customs, cultural performances, traditional celebrations, religious events and temple festivals
**Recreation and Tourism Resources**	Not tourism attractions per se, but tourism facilities set up for leisure and recreation		Playgrounds, sports complexes, resorts

Note: Tourism resources are divided with reference to <Classification, investigation and evaluation of tourism resources> (GB/T 18972–2017), <Tourism Scenic Area Classification> (T/CTAA 0001–2019) and <2018 China Tourism Scenic Area Development Report> (Department of Resource Development, Ministry of Culture and Tourism, China, 2018).

#### 3.2.3 Geodetector model

The Geodetector is a method for measuring spatial heterogeneity and explaining its influencing factors. It can be used to identify and examine regional variations and evaluate the influence of independent variables on dependent variables by revealing their similarities in spatial distribution [62,63]. This research method has been applied in multiple fields of research; in contrast to general regression models, the Geodetector avoids the collinearity problem in the independent variables [62]. The Geodetector consists of four main parts, one of which is factor detection, which can be used to determine the spatial differentiation of Y or detect the extent to which factor X explains the spatial variation of attribute Y. As the density value clearly reflects its spatial dispersion or agglomeration characteristics and the process of change of this pattern [1], the kernel density of tourists’ visits to attractions during 2014–2021 was selected as the dependent variable (y) in this study, and the impact of the influencing factor indicator (X) on the spatial distribution of tourists’ perceived attractions was detected through a factor detector, after which the results were measured by the q-value (with a range of [0, 1]). This value represents how the independent variable explains 100 × q% of the dependent variable. In this case, the larger the q-value [62], the greater the impact of the spatial distribution of tourists’ perceived attractions. This was calculated as follows:

$$q=1\;-\;\frac{\sum_{h=1}^{L}N_{h}\sigma_{h}^{2}}{N\sigma^{2}}$$ (1)

In this formula, h = 1, 2, 3…, L, where h denotes the sub-areas, L denotes the number of sub-areas and *N*_*h*_ denotes the number of samples in the h-th (h = 1, 2, 3…, L) study area. Additionally, N denotes the number of samples in the entire study area, while $\sigma_{h}^{2}$ and *σ*^2^ are the variances of Y values in the h-th (h = 1, 2, 3…, L) study area and in the entire study area, respectively.

## 4. Results

This section details the changes in tourists’ perceptions of Dali Prefecture’s attractions over the eight-year period of 2014–2021 and visualizes the distribution and perception of attractions through ArcMap 10.8 to clarify the spatial distribution characteristics. Factor detection in the Geodetector model is used to evaluate whether the influencing factor indicators affect tourists’ perceptions of the attraction and the process of change in q-values over the eight-year period.

### 4.1 Analysis of the perceived degree of different tourism resources

This study categorized the 271 attractions obtained according to the tourism resource categories. The number of tourist resources and reviews from 2014–2021 is shown in Table 3. For the purpose of investigating the differences in the perceptions of tourism resources at different periods, we divided the time into four periods, categorized the data based on the release date and evaluated the perceived degree of the attractions according to the number of reviews.

**Table 3:** Analysis of the tourism resources and number of reviews in different periods.

Category		Segment Types	2014–2015 (Period 1)			2016–2017 (Period 2)			2018–2019 (Period 3)			2020–2021 (Period 4)			Total		
			att	rev	%	att	rev	%	att	rev	%	att	rev	%	att	rev	%
**Natural Resources**		Physiography	9	488	10.9	11	1699	14.0	13	1919	19.0	12	1685	17.2	13	5791	15.9
		Waters	9	411	9.2	23	1430	11.8	23	901	8.9	25	1331	13.6	30	4073	11.2
		Biological	2	4	0.1	4	22	0.2	5	25	0.2	4	26	0.3	6	77	0.2
		**Subtotal**	**20**	**903**	**20.2**	**38**	**3151**	**26.0**	**41**	**2845**	**28.2**	**41**	**3042**	**31.1**	**49**	**9941**	**27.3**
**Humanistic Resources**	With historical value	Historic sites	6	596	13.3	10	1273	10.5	11	656	6.5	8	652	6.7	12	3177	8.7
		Ancient villages and towns	9	268	6.0	15	750	6.2	14	329	3.3	16	280	2.9	18	1627	4.5
		Architecture and facilities	11	113	2.5	30	502	4.1	36	352	3.5	36	366	3.7	44	1333	3.7
		Religious sites	17	462	10.3	43	1374	11.3	47	1757	17.4	37	1980	20.2	58	5573	15.3
		Historic gardens	7	823	18.4	12	2312	19.1	12	1814	18.0	13	1967	20.1	13	6916	19.0
		**Subtotal**	**50**	**2262**	**50.5**	**110**	**6211**	**51.3**	**120**	**4908**	**48.6**	**110**	**5245**	**53.6**	**145**	**18626**	**51.1**
	Uncertain historical value	Cultural venues	2	32	0.7	5	108	0.9	9	66	0.7	14	100	1.0	14	306	0.8
		Modern gardens	0	0	0.0	2	39	0.3	4	34	0.3	4	26	0.3	4	99	0.3
		Theme park	1	178	4.0	1	684	5.6	1	650	6.4	1	293	3.0	1	1805	4.9
		Featured street	2	26	0.6	2	98	0.8	6	62	0.6	8	50	0.5	8	236	0.6
		Rural landscape	12	769	17.2	26	1353	11.2	24	616	6.1	29	486	5.0	35	3224	8.8
		**Subtotal**	**17**	**1005**	**22.5**	**36**	**2282**	**18.8**	**44**	**1428**	**14.1**	**56**	**955**	**9.8**	**62**	**5670**	**15.5**
	Human Activities		1	233	5.2	3	321	2.7	3	732	7.2	6	271	2.8	7	1557	4.3
**Recreation and Tourism Resources**			3	73	1.6	6	144	1.2	6	191	1.9	8	267	2.7	8	675	1.9
**Total**			**91**	**4476**	**100.0**	**193**	**12109**	**100.0**	**214**	**10104**	**100.0**	**221**	**9780**	**100.0**	**271**	**36469**	**100.0**

Note: Period 1 corresponds to comments from 1 January 2014 to 31 December 2015; Period 2 corresponds to comments from 1 January 2016 to 31 December 2017, etc.

Att: Number of attractions; Rev: Number of reviews.

Based on the results, the tourists in Dali Prefecture had the highest degree of perception for humanistic resources with historical value (51.1%), followed by natural resources (27.3%) and humanistic resources with uncertain historical value (15.5%). Conversely, their perception of human activities (4.3%) and recreation and tourism resources (1.9%) was low.

In terms of time, their perception of natural resources during the four time periods was 20.2%, 26.0%, 28.2%, and 31.1%, indicating an increasing trend. Additionally, the perception of humanistic resources with historical value was 50.5%, 51.3%, 48.6%, and 53.6%, respectively; indicating a temporary decline, but an overall increase. In contrast, the perception of humanistic resources with uncertain historical value declined from 22.5% to 9.8%. This indicates that their interest in natural and history-related humanistic resources increased over time. Meanwhile, religious sites showed an increasing trend from 10.3% to 20.2%, while the perception for rural landscapes decreased from 17.2% to 5.0%.

### 4.2 Comparison of spatial distribution among tourists’ perceived tourism resources

**Fig 3:**
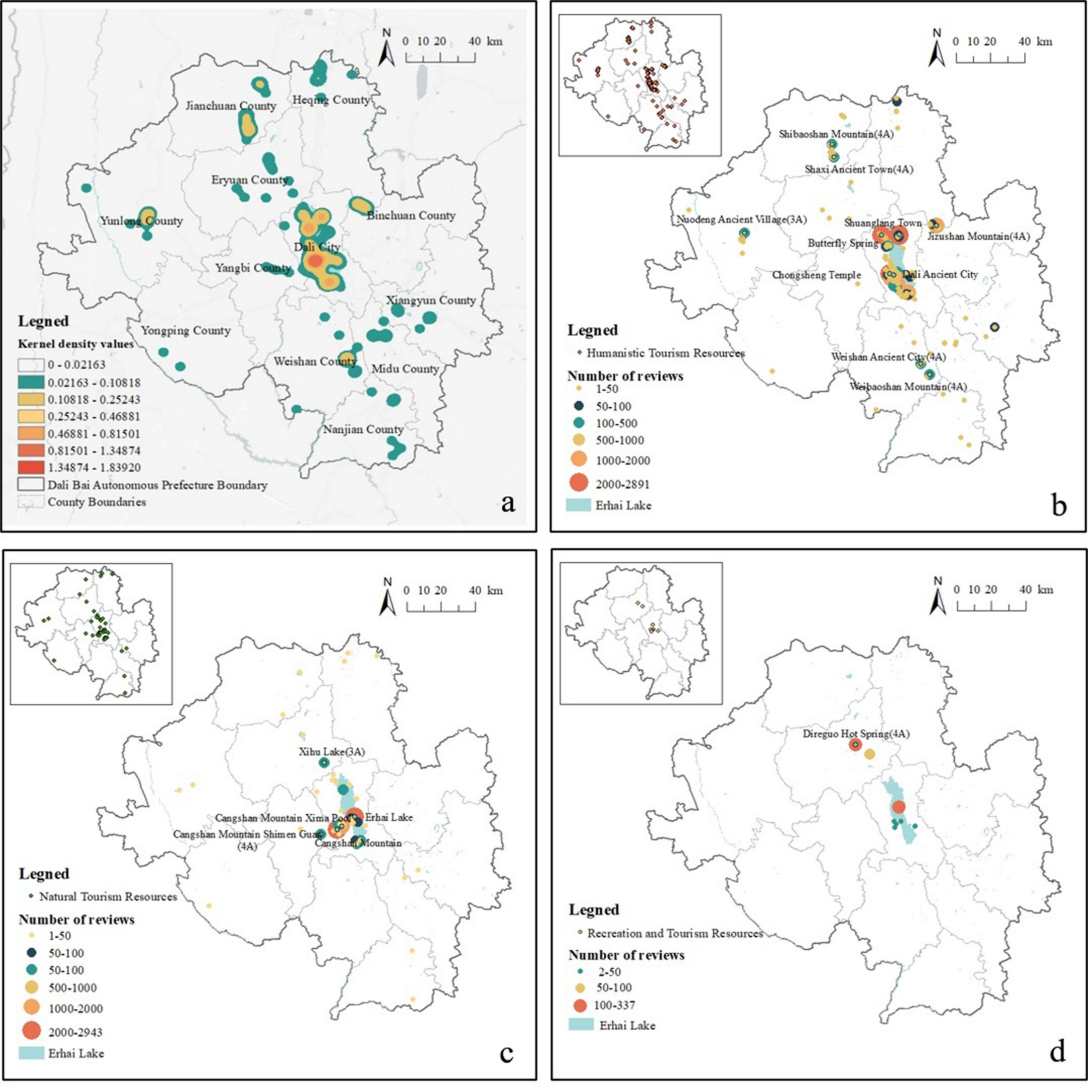
Spatial distribution of tourists’ perceived tourism resources. Notes: a. Kernel density map of tourists’ perceived tourism resources; b. Perceived degree of humanistic resources; c. Perceived degree of natural resources; d. Perceived degree of recreation and tourism resources. The circle in the graph represents the number of reviews, i.e. the larger the circle, the more reviews. The graph in the upper-left corner indicates the geospatial distribution of different types of tourism resources. The base map images are from the China Ministry of Natural Resources(http://bzdt.ch.mnr.gov.cn/index.html). The drawing approval number is GS (2016)1593.

To understand the spatial distribution of visitor-perceived attractions, we visualized the data and created a kernel density map (Fig 3A). The results indicated that the attractions perceived by the tourists in Dali Prefecture were mainly concentrated in Dali City, and in Jianchuan, Weishan, Binchuan and Yunlong Counties.

#### 4.2.1 Humanistic resources

According to the distribution of landscape resource types and perceived degree, there were 214 attractions in the humanistic resource category, accounting for 78.9% of the total attractions (Fig 3B). Additionally, the tourists had a relatively high perception of certain attractions in each county. For instance, the highest perceived level (1,094 reviews) was for Jizushan Mountain (a religious site) in Binchuan County, followed by Shaxi Ancient Town (534 reviews) and Shibaoshan Mountain (300 reviews) in Jianchuan County. As a Buddhist shrine in Southeast Asia [55], Jizushan Mountain receives a constant stream of tourists. Meanwhile, due to the inconvenience of transportation, Shaxi Ancient Town has been excluded from the momentum of development; the local ethnic characteristics were thus well preserved.

Located a few kilometers away from Shaxi Ancient Town is Shibaoshan Mountain, one of the major scenic attractions in China. Upon visiting this site, Fei Xiaotong (a well known sociologist, anthropologist, ethnographer, social activist and one of the founders of Chinese sociology and anthropology) exclaimed that “there are Dunhuang murals in the north and Jianchuan Grottoes in the south” [64]. After Shibaoshan Mountain, there is Weishan Ancient City (179 reviews) and Weibaoshan Mountain (175 reviews) in Weishan County.

The perceived degree of the ancient village of Nuodeng in Yunlong County (141 reviews) was also relatively high. Nuodeng was the earliest location of the Wujing Salt Division (was established during the Ming Dynasty with its headquarters in Dali Prefecture, where it was in charge of salt production, taxation, transportation and marketing.)during the Ming Dynasty [65]. Conversely, the perception of other attractions in the counties (under 100 reviews) generally decreased, along with the perception of human activities.

#### 4.2.2 Natural resources

In this study, there were 49 attractions in the natural resources category, accounting for 18.1% of the total attractions. They were mainly concentrated in Dali City (Fig 3C), with the most representative ones being Cangshan Mountain (2,943 comments), Erhai Lake (2,816 comments) and Xima Pool (1,928 comments).

#### 4.2.3 Recreation and tourism resources

Recreation and tourism resources were mainly distributed in Dali City and Eryuan County (Fig 3D), where the highest perceived degree was Dali Direguo (hot springs) (337 reviews). However, the perceived number of attractions was low, compared to natural and humanistic resources.

### 4.3 Evaluation of the spatial perception of influencing factors

#### 4.3.1 Analysis of the influencing factors

The results showed that the kernel density of the 10 selected influencing factors and attractions in this study passed the significance test at the 0.05 level, indicating that the factors had significant differences in the spatial distribution of the attractions. The q-values of the influencing factor results were also used to explain the explanatory power of the factors in the spatial distribution of attractions.

According to the findings (Table 4), socioeconomic factors had the highest explanatory power. Specifically, the highest explanatory power (q-value) was found in the proportion of the tertiary industry X_8_ (0.583), followed by the GDP X_7_ (0.513) and the resident population X_6_ (0.450). Moreover, the proportion of the tertiary industry represents the level of the development of the regional service industry [1,15], while the resident population determines the scale of the local tourism market, indicating that the level of regional economic development and tourism services are the key influencing factors in tourists’ visits to attractions [1].

**Table 4:** Influencing factors’ q-value results.

Year	Traffic Accessibility			Natural Environment		Socioeconomic Factors			Core Resources	
	X_1_	X_2_	X_3_	X_4_	X_5_	X_6_	X_7_	X_8_	X_9_	X_10_
2014–2015	0.256	0.376	0.399	0.133^*^	0.276	0.386	0.359	0.494	0.264	0.273
2016–2017	0.241	0.310	0.427	0.220	0.265	0.432	0.466	0.513	0.264	0.143
2018–2019	0.201	0.406	0.607	0.139	0.193	0.480	0.634	0.671	0.270	0.194
2020–2021	0.177	0.423	0.499	0.128	0.230	0.502	0.592	0.656	0.314	0.173
Average value	0.219	0.379	0.483	0.155	0.241	0.450	0.513	0.583	0.310	0.196

Note: * indicates significance at the 5% level (p < 0.05); unmarked * indicates significance at the 1% level (p < 0.01).

The explanatory power of traffic accessibility was also high, as shown in the average q-values of the distance from a city X_3_ (0.483) and from a high-speed rail station X_2_ (0.379). This indicates that tourists are strongly influenced by these aspects when visiting attractions. Conversely, the relatively low mean q-value of distance from a high-level highway X_1_ (0.219) indicates that tourists are less dependent on it when visiting attractions.

In addition, the explanatory power of core resources was relatively low. Among them, the average q-value of the distance from high-level attractions X_9_ (0.310) was high, indicating that tourists are influenced by the presence/absence of nearby high-level attractions when visiting attractions. In contrast, the average q-value of the distance from humanistic resources X_10_ (0.196) was lower than the other factors, indicating that tourists are less dependent on them when visiting attractions.

Regarding the natural environment factors, they had weak explanatory power on tourists visiting attractions. Specifically, the average q-values for the distance from waters X_5_ and the altitude of attraction X_4_ were 0.241 and 0.155, respectively, indicating that tourists are less dependent on these aspects when visiting attractions.

#### 4.3.2 Changes in the influencing factors during different periods

**Fig 4:**
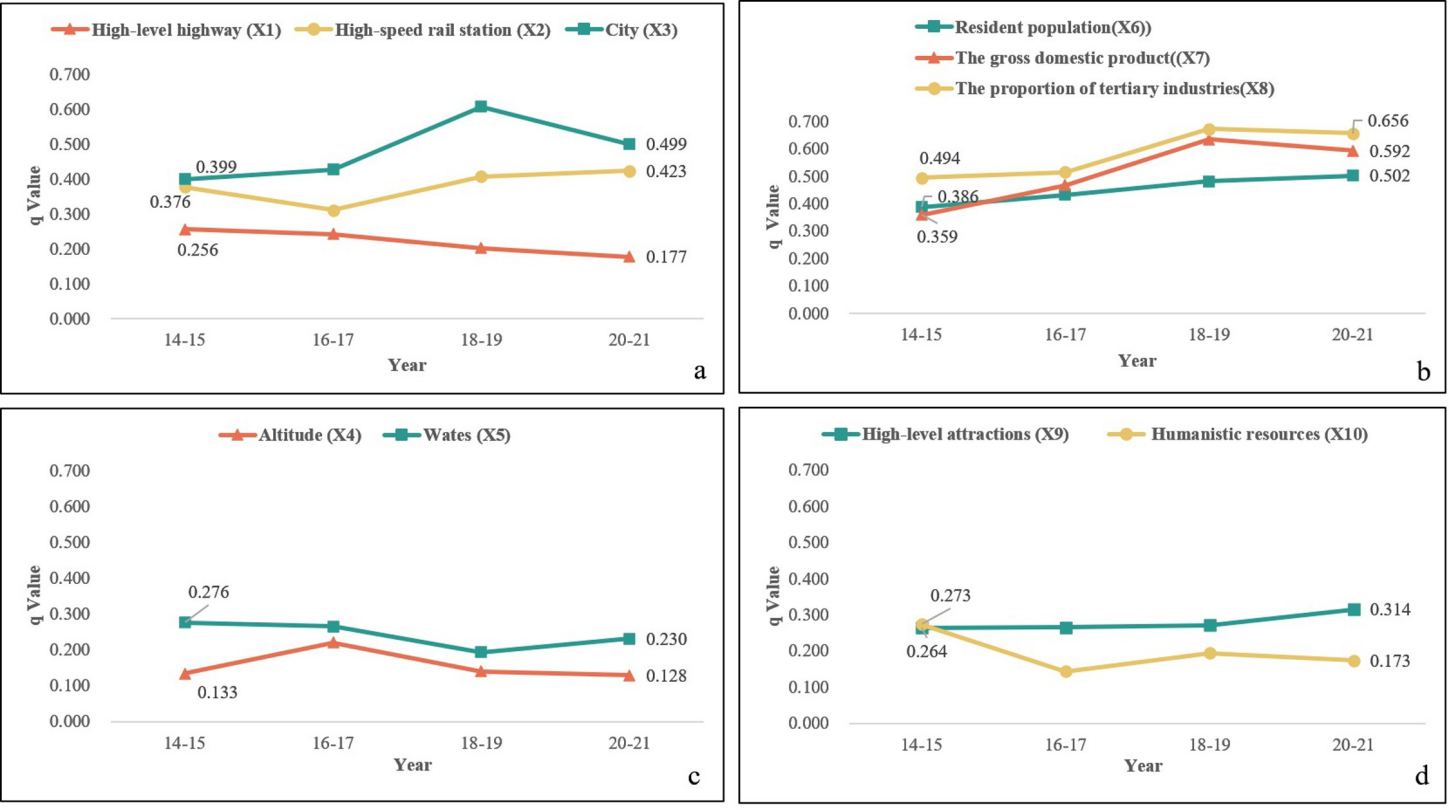
Changes in the influencing factors during different periods. Notes: a. Change in the q-value of traffic accessibility; b. Change in the q-value of socioeconomic factors; c. Change in the q-value of the natural environment; d. Change in the q-value of core resources.

As shown in Fig 4, the influence of each indicator on the spatial distribution of tourists’ perceived attractions in Dali Prefecture from 2014 to 2021 varied.

As for traffic accessibility, the q-value of the distance from a high-level highway (X_1_) decreased from 0.256 to 0.177, whereas that of the distance from a high-speed rail station (X_2_) increased from 0.376 to 0.423. This indicates the decreasing influence of the distance from a high-level highway on tourists visiting attractions and a growing dependence on high-speed rail stations. Additionally, the q-value of the distance from a city (X_3_) increased from 2014–2019 (with a decrease from 2020–2021), indicating that the distance from a city had an increasing influence on tourists visiting attractions.

Among the socioeconomic factors, the q-values of resident population, the GDP and the tertiary industry increased from 2014 to 2019, but declined from 2020 to 2021. However, the overall trend was on the rise, indicating that the degree of influence by the level of regional economic development and tourism services on tourists visiting attractions increased.

Regarding the natural environmental factors, the q-value of altitude (X_4_) showed an increasing trend from 2014 to 2017, but a decrease from 2018 to 2021. Specifically, the q-value of waters (X_5_) decreased from 2014 to 2019 and increased from 2020 to 2021. However, these changes were not significant.

As for the core resources, the q-value of high-level attractions increased from 0.264 to 0.314, indicating that tourists were gradually influenced by such attractions when visiting Dali Prefecture. In contrast, the q-value of humanistic resources was the highest in 2014–2015, after which there was an overall decrease.

## 5. Discussion

Traditional spatial perception studies rely on subjective human evaluations and can only investigate small-scale site perceptions. The development of big data has provided new data support, which, combined with the use of new technologies, has made it possible to analyze tourist perceptions on a large scale. However, as mentioned previously, there is a lack of research on spatial perception in remote minority areas. Based on previous research, we combined Internet data and the Geodetector model to evaluate spatial perceptions of tourist attractions in remote minority areas. Compared to traditional regression models, Geodetector avoids the problem of collinearity due to the strongly correlated independent variables. In this section, we elaborate on the characteristics of tourists’ perceptions of tourism resources in Dali Prefecture and their spatial distribution, highlight the differences in the explanatory power of the influencing factors, and suggest limitations of the current work and directions for future work in light of previously conducted research.

### 5.1 Tourists’ perceived characteristics of tourism resources in Dali Prefecture

Our findings indicated that tourists’ perception of humanistic resources with historical value was the highest, followed by natural resources. Conversely, the perception of humanistic resources with uncertain historical value decreased. Some studies [66–68] have indicated that typical cultural and natural resources are the primary motivations for tourists to take a trip. People tend to travel when they encounter trouble or failure and want to escape from daily life for a short time period [69]. Historical and cultural resources provide visitors with a temporary escape from the pressures of daily life, while satisfying their search for nostalgia and peace of mind [70]. Natural resources can also give tourists a sense of peace and tranquility, contributing to their psychological well-being and make the trip more pleasant [71].

In addition, we found that an increasing number of tourists have become interested in Dali’s religious culture. Religion has existed in China for thousands of years and is an irreplaceable part of Chinese culture [72]. Previous studies on Chinese tourism found that temples are the most popular tourist destinations; they draw a large number of visitors who want to experience local history, lifestyles, traditions and folk art [67,73]. Specifically, Dali has been at the intersection of Theravada, Tibetan, Indian and Chinese Central Plains Buddhism since the Nanzhao period [20]. Conversely, tourists’ perception of an idyllic landscape continues to decline, probably due to the loss of the authenticity of rural landscapes. According to previous studies, today’s tourists are eager to find ‘unspoiled, pristineness, genuineness and traditional’ culture as a way to seek peace of mind [67,74].

### 5.2 Comparison of the spatial distribution of tourists’ perceived tourism resources

The tourism resources perceived by tourists to be high were mainly concentrated in the Dali City, rather than the surrounding counties. This can be explained by the fact that the city of Dali is more accessible than the surrounding counties, and the various governmental bodies within its jurisdiction have begun marketing the local historical and cultural resources and iconic attractions to increase the attractiveness of the attractions, and Zhao’s findings are consistent with this [54]. Moreover, the city has been designated as a “National Scenic Area” and a “National Nature Reserve” by the State Council and as one of the “Top 10 Most Attractive Cities in China” by China Central Television [54].

In contrast, the counties have relatively poor traffic accessibility; however, the tourists have a relatively high degree of perception of certain attractions. Some studies have found that tourists tend to visit high-visibility attractions regardless of distance [28–30]. Furthermore, we found that attractions with a higher degree of perception in each county were popular and were 4A or 3A grade tourist attractions (Fig 3). The tourism infrastructure of A-grade tourist attractions provides tourists with a better experience than non-A-grade attractions. Ultimately, tourists decide to visit an attraction based on its attractiveness and traffic accessibility [75].

### 5.3 Differences in the explanatory power of the influencing factors on spatial perception

Our results showed that tourists’ spatial perceptions of tourism resources were related to 10 influencing factors, with differences in the explanatory power of each factor. Among them, socioeconomic factors had the strongest explanatory power, indicating that the level of tourism development had an increasing influence on tourists visiting attractions [15]. Additionally, the GDP and the proportion of the tertiary industry showed consistent growth between 2014 and 2021. However, there was a significant decrease in 2020–2021 due to the coronavirus pandemic in January 2020. Since then, China has implemented a strict prevention policy, which has greatly impacted the tourism industry.

Despite its rich natural environment and tourism resources, Yunnan’s tourism development has experienced long-term constraints, due to its mountainous geography and poor transportation conditions [76]. The results indicated that accessibility has a strong influence on how easily tourists can access their destinations [77]. Our analysis revealed that tourism transportation in Dali Prefecture changed from primarily road transportation to high-speed rail transportation. This is contrary to Su’s finding that “tourism in Yunnan Province is more dependent on road passenger transportation” [24]. In addition, because cities are the main locations where visitors tend to spend time, the distance between them and the attractions is an important influencing factor. Visiting attractions in peripheral urban areas implies long journeys and requiring multiple means of transport, decreasing the desire to visit such locations. Thus, the development of effective and reliable transport connections is key to promoting tourism in peripheral urban areas [78].

Regarding core resources, their explanatory power was relatively low, and the tourists were more influenced by the presence or absence of nearby high-level attractions. Conversely, as the tourists did not pay enough attention to humanistic resources, raising awareness of such resources (e.g. national cultural heritage protection units and traditional villages) is necessary to enhance their attractiveness to tourists.

Finally, natural environment factors had a low explanatory power. This was likely due to the mountainous geography of Dali Prefecture, where changes in the waters and altitude were not obvious during the short research period; therefore, the tourists visiting the attractions were not significantly affected by these factors. As stated in previous research, tourist attractions not only depend on a good natural environment but also require subsequent development and construction [1].

### 5.4 Limitations and future research

This study includes some limitations that should be noted. First, in terms of data, the results cannot be generalized because there may be some differences in landscape preferences among the different age groups. Second, we analyzed the coordinate data of the attractions, which did not cover a wide enough area. Hence, future research should analyze text and photo data to further grasp tourists’ perceptions of tourism resources in Dali Prefecture. Third, the sample in this study was primarily Chinese tourists and did not consider individuals from other cultures. Therefore, future studies should include a wider demographic to generalize the findings.

## 6. Conclusion

With tourism playing an increasingly important part in people’s lives, understanding the current state of tourists’ perceptions of regional tourism is crucial to promoting the development of the tourism industry. In this study, we constructed a research framework for spatial perception measurement of remote minority areas by collecting Internet reviews data, using spatiotemporal data calculation and the Geodetector model, and quantified the strength of the explanations of the influencing factors for tourist visits to attractions. We took Dali Prefecture as the study area and analyzed the degree of tourists’ perceptions of Dali Prefecture’s attractions, the spatial distribution of tourists’ perceived attractions, and the process of change in the explanatory power of their influencing factors over an eight-year period.

The results indicated that tourists had the highest perception of human resources (attractions) with historical value in Dali Prefecture and were mainly influenced by tourism infrastructure, attractiveness, and traffic accessibility when visiting attractions. Although Dali Prefecture has the advantages of a good natural environment, rich historical and cultural resources, and a longstanding religious culture, it continues to face the problem of uneven tourism development. The results validate the feasibility of the research methodology and confirm the uneven development of tourism in Dali Prefecture. The findings of the study can guide policy makers and government departments to make effective decisions on tourism planning in Dali, thus contributing to the sustainable development of tourism there. Meanwhile, this study provides a method for the study of perception in remote areas in a large scale and over a long period of time, expanding the research perspective on spatial perception.

## Supporting information

S1 File Results of Geodetector. (ZIP)

S2 File Data on coordinates of attractions. (XLS)

S3 File Detailed data on attractions. (XLSX)
